# Cigarette smoke worsens lung inflammation and impairs resolution of influenza infection in mice

**DOI:** 10.1186/1465-9921-9-53

**Published:** 2008-07-15

**Authors:** Rosa C Gualano, Michelle J Hansen, Ross Vlahos, Jessica E Jones, Ruth A Park-Jones, Georgia Deliyannis, Stephen J Turner, Karen A Duca, Gary P Anderson

**Affiliations:** 1 Department of Pharmacology, The University of Melbourne, Parkville 3010, Victoria, Australia; 2 Department of Medicine, The University of Melbourne & The Royal Melbourne Hospital, Parkville 3050, Victoria, Australia; 3 Department of Microbiology & Immunology, The University of Melbourne, Parkville 3010, Victoria, Australia; 4 Co-Operative Research Centre for Chronic Inflammatory Diseases, Parkville 3052, Victoria, Australia; 5 Virginia Bioinformatics Institute, Blacksburg, Virginia, USA

## Abstract

**Background:**

Cigarette smoke has both pro-inflammatory and immunosuppressive effects. Both active and passive cigarette smoke exposure are linked to an increased incidence and severity of respiratory virus infections, but underlying mechanisms are not well defined. We hypothesized, based on prior gene expression profiling studies, that upregulation of pro-inflammatory mediators by short term smoke exposure would be protective against a subsequent influenza infection.

**Methods:**

BALB/c mice were subjected to whole body smoke exposure with 9 cigarettes/day for 4 days. Mice were then infected with influenza A (H3N1, Mem71 strain), and analyzed 3 and 10 days later (d3, d10). These time points are the peak and resolution (respectively) of influenza infection.

**Results:**

Inflammatory cell influx into the bronchoalveolar lavage (BALF), inflammatory mediators, proteases, histopathology, viral titres and T lymphocyte profiles were analyzed. Compared to smoke or influenza alone, mice exposed to smoke and then influenza had more macrophages, neutrophils and total lymphocytes in BALF at d3, more macrophages in BALF at d10, lower net gelatinase activity and increased activity of tissue inhibitor of metalloprotease-1 in BALF at d3, altered profiles of key cytokines and CD4+ and CD8+ T lymphocytes, worse lung pathology and more virus-specific, activated CD8+ T lymphocytes in BALF. Mice smoke exposed before influenza infection had close to 10-fold higher lung virus titres at d3 than influenza alone mice, although all mice had cleared virus by d10, regardless of smoke exposure. Smoke exposure caused temporary weight loss and when smoking ceased after viral infection, smoke and influenza mice regained significantly less weight than smoke alone mice.

**Conclusion:**

Smoke induced inflammation does not protect against influenza infection.

In most respects, smoke exposure worsened the host response to influenza. This animal model may be useful in studying how smoke worsens respiratory viral infections.

## Background

Cigarette smoke exposure is a major but preventable cause of increased risk of lung infections in children and adults. Smoke exposure is linked in adults to an increased incidence and severity of asthma, impaired lung function and airway inflammation. Exposure to cigarette smoke has similar adverse effects to active smoking on lung infections, with young children at higher risk [1,2]. Nearly half of the world's children breathe cigarette smoke at home [1], and both pre- and post-natal smoke exposure are linked to decreased lung function and increased risk and severity of asthma and respiratory infections [1,2]. In comparison to nonsmokers, smokers are at greater risk of acquiring symptomatic common colds [3] and smokers had greater infection rates and disease severity during an influenza epidemic [4].

Influenza (flu) is a common and potentially serious viral infection at all ages. It is caused by the influenza virus, which has a segmented, negative strand RNA genome and belongs to the *Orthomyxoviridae *family. Over three recent successive winters in the UK, a total of 32% of patients seeing a doctor with symptoms of respiratory infection had PCR-detected influenza [5]. The role of inflammation in the resolution of influenza infection remains controversial, with evidence for both immune-mediated amelioration [reviewed in [6]] and worsening [7-9] of the host's overall condition.

Cigarette smoke has both immune activating and suppressing activities [10] which can persist after smoking cessation [11]. The effects of short term smoke exposure on the acquisition and course of a respiratory virus infection, which reflects some of the incidental community exposure to smoke, are not well understood. We and others have previously described mouse models where smoke exposure increases expression of factors likely to be important in antiviral defence [12,13]. These factors include key innate immune mediators and chemokines such as tumor necrosis factor-α (TNF-α), macrophage inflammatory protein-2 (MIP-2), monocyte chemoattractant protein-1 (MCP-1) and interleukin-6 (IL-6). As human influenza strains can be used to infect mice, and influenza pathology in mice is similar to humans [6,14], we chose a mouse model to study the effect of cigarette smoke on host responses to influenza.

In this current study, we hypothesized that the induction of such host defence factors and inflammatory mediators in smoke exposed mice would largely protect against harmful pathology from subsequent influenza infection. However, we found that short term smoke exposure before influenza infection led to an increase in most aspects of inflammation, higher viral titres and greater loss of body weight. Our results may improve the understanding of how smoke exposure worsens respiratory virus infections.

## Methods

### Mice

Specific pathogen-free male BALB/c mice were obtained from the Animal Resource Centre (Perth, Australia) at age 6–8 weeks. BALB/c mice were used as we have previously shown them to be the most susceptible strain to smoke-induced inflammation [12] and they are suitable for work with influenza [6], including tetramer staining. Mice were housed at 20°C on a 12-hr light/dark cycle in sterile microisolator cages and fed *ad libitum *with sterile chow and water. All mouse procedures were approved by the University of Melbourne Animal Experimentation Ethics Committee and complied with the standards of the National Health and Medical Research Council of Australia. After arrival, mice were acclimatized for 3 days.

### Virus and cell culture procedures

The intermediate virulence H3N1 (Mem71) strain of influenza A is a genetic reassortant of A/Memphis/1/71 (H3N2) × A/Bellamy/42 (H1N1) [15]. The virus was grown in MDCK cells, which were maintained at 37°C/5% CO_2 _in RPMI 1640 plus 2 mM L-glutamine, 2 mM sodium pyruvate, 30 μg/ml gentamycin, 100 I.U./ml penicillin, 100 μg/ml streptomycin and 10% v/v heat inactivated FCS (all from Invitrogen). To grow virus stocks, 90% confluent MDCK cells were washed twice in PBS and infected with egg grown virus at 0.01 plaque forming units (pfu)/cell, or mock infected with medium alone. After 1.5 hr inoculum was removed, cells were washed twice with PBS and low protein, serum free medium (VP-SFM, Invitrogen) containing 0.5 μg/ml trypsin was added. Virus was harvested 48 hr later, when there was extensive viral cytopathic effect. Flasks were shaken to loosen cells, the medium and cells were collected, briefly vortexed, subjected to a low speed clearing spin and the clarified supernatant stored in aliquots at -80°C.

Virus was quantitated by plaque assay in MDCK cells, which were seeded at 4 × 10^6 ^cells per 6-well plate and used the next day. Cells were washed twice in PBS, then 150 μl of virus diluted in serum-free RPMI was added to duplicate wells. Plates were incubated at 37°C on a rocker for 1 hr. Cells were overlaid with 3 ml per well of an equal mix of double strength, serum free Leibovitz L15 medium (Invitrogen) and 1.8% agarose (Sigma A-6013) prepared in water (both held at 45°C). After 2 days at 37°C, cells were fixed with 5% formaldehyde in saline for 4 hr, the agarose was removed and cells stained for 45 min with 0.5% crystal violet (Sigma) in methanol. Plates were rinsed in water and plaques counted.

### Cigarette smoke exposure & virus infection

Mice were subjected to whole body exposure of the smoke of 9 Winfield Red cigarettes per day (delivered as 3 cigarettes, 3 times/day) for 4 days, as previously described [12]. This smoke exposure protocol causes acute inflammation, but not acute lung injury and plasma corticosterone analysis indicated that mice are not unduly stressed [16]. As nicotine reduces appetite, leading to weight loss in smoke exposed mice, all mice were weighed daily during smoke exposure and most days thereafter. The 4 groups in this study were no treatment, influenza (flu) alone, smoke alone, and smoke and (then) influenza. There was no further smoke exposure after influenza infection.

Mice destined for influenza infection were anaesthetized by penthrane inhalation (Medical Developments Australia) and infected intranasally with 10^4.5 ^plaque forming units of influenza in a 50 μl volume, diluted in serum free, low protein medium (VP-SFM, Invitrogen). All mice, including the no treatment and smoke alone groups, were dissected at day 3 (peak) and day 10 (resolution) after the influenza infection (d3, d10).

### Dissection of mice

Mice were killed by an intraperitoneal ketamine/xylazine overdose and bronchoalveolar lavage (BALF) collected as previously described [12]. Lungs destined for virus titration or histology were not lavaged [17]. To determine influenza virus titres in lungs, the entire lungs were removed, weighed and homogenized briefly in serum-free RPMI containing 30 μg/ml gentamycin, 100 I.U./ml penicillin and 100 μg/ml streptomycin. Clarified homogenate was snap frozen, stored at -80°C and used in plaque assays. Viable cells in BALF were counted by fluorescence microscopy [18] and cytospins prepared using 200 μl BALF spun at 350 rpm for 10 min on a Cytospin 3 (Shandon, UK). BALF was briefly centrifuged to pellet cells, the pellet was saved for flow cytometry and the clarified BALF stored at -80°C for ELISAs and protease assays. Cytospin slides were stained with DiffQuik (Dade Baxter, Australia) and at least 500 cells per slide were differentiated into eosinophils, neutrophils, lymphocytes and macrophages by standard morphological criteria [19].

### Protease expression and activity in BALF

Total gelatinase activity of non-concentrated, pooled BALF was assayed by zymography, and net gelatinase activity in BALF assayed by digestion of fluorescein coupled gelatin, as previously described [12]. Densitometry of zymography gels was done using Kodak 1D Image Analysis Software. Net serine protease activity in BALF was assayed as previously described [12].

### Flow Cytometry

Cells from BALF pellets and mediastinal (draining) lymph nodes (LNs) were pooled for each treatment group of 8 mice, and used at once without culture or stimulation. Lymphocytes were purified from LNs using Lympholyte (Cedarlane, Canada) as specified by the manufacturer. BALF pellets were stained directly. All antibodies were from PharMingen. Cells were washed in and then resuspended in 1 ml FACS buffer (PBS + 1% FCS) and counted. Samples from influenza infected mice contained the most cells and were diluted to 250,000 cells for each staining reaction. All other samples were used undiluted. Cells were stained for the constitutive markers CD3, CD4 and CD8 and the activation markers CD25 (d3 only) or CD44 (d10 only). With d10 samples, influenza specific lymphocytes were stained with 10 μg/ml of the phycoerythrin (PE)-coupled Kd NP 147–155 tetramer (sequence TYQRTRALV) and counterstained with peridinin chlorophyll protein (PerCP)-coupled CD8. Cells were incubated with antibody for 45 min at 4°C, washed twice in and resuspended in FACS buffer and paraformaldehyde (PFA) was added to a final concentration of 1%. Data were acquired on a BD FACSCalibur, gated on lymphocytes by standard forward and side scatter properties, and set to acquire 10,000 gated events. As many gated events as possible were acquired with no treatment mice, but this was <10,000 with BALF stains. BD CELLQuest software was used for acquisition and analysis. Results are expressed as number of that type of cell in the pooled sample of 8 mice.

### Real time PCR

Lungs were perfused free of blood with PBS, removed and snap frozen. Extraction of total lung RNA using RNeasy kits (Qiagen), reverse transcription with SuperScript III (Invitrogen) and triplicate real time PCR reactions with Applied Biosystems pre-developed assay reagents and 18S rRNA internal control were done as previously described [12]. Tissue pieces of equivalent size from lungs of the 4 mice (allocated for real time PCR and therefore not lavaged) in each treatment group were pooled prior to RNA extraction. Relative quantities of mRNA for target genes is expressed as a fold difference in comparison to no treatment mice.

### ELISAs

Tissue inhibitor of metalloprotease-1 (TIMP-1), macrophage inflammatory protein-2 (MIP-2), monocyte chemotactic protein-1 (MCP-1), interleukin-4 (IL-4), tumor necrosis factor-α (TNF-α), granulocyte-macrophage colony stimulating factor (GM-CSF) and interferon-γ (IFN-γ) in BALF were quantitated using R&D Systems or PharMingen kits as per manufacturer's instructions. Pooled samples from the 8 mice in each group were assayed in duplicate. IFN-α/β in BALF were quantitated by bioassay [20].

### Histology

Mouse lungs were perfusion fixed *in situ *via a tracheal cannula with 4% PFA at 200 mm H_2_O pressure. After 10 min, the trachea was ligated, the lungs were left *in situ *for 1 hr, then removed and immersed in 4% PFA for at least 24 hr. After fixation of the lung tissue and processing in paraffin wax, sections (3 – 4 μm thick) were cut and stained with hematoxylin and eosin (H&E). Slides were viewed on a Nikon E600 microscope and photographed at 200× magnification with a Nikon DXM1200 camera running from ACT-1 software.

### Statistics

Data were analyzed and graphed using GraphPad PRISM version 4.0. Data were generally analyzed using two-way ANOVA, and when significance was achieved, a Bonferroni post hoc test was used. Body weight comparisons were analyzed using ANOVA with repeated measures, followed by a post hoc Fisher's protected least significance difference test. Viral titres in lung were compared using an unpaired Student's two-tailed t-test. Unless otherwise stated data are presented as mean +/- standard error of mean (SEM) for at least 8 mice/group. Probability values less than 0.05 were considered significant. The symbol * indicates a significant change (p < 0.05); ** p < 0.01, ***p < 0.001, by ANOVA and Bonferroni test.

## Results

### Weight changes

**Figure 1:**
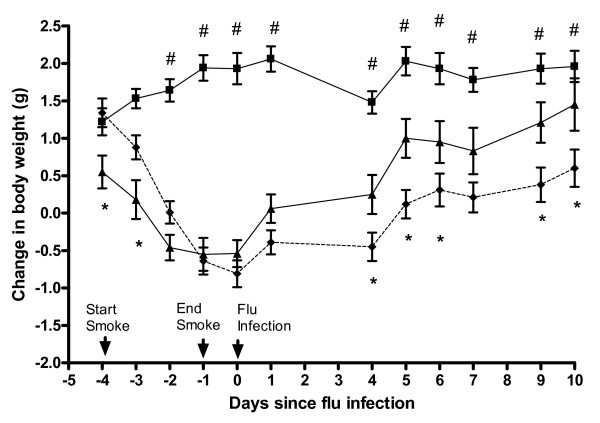
**Change in body weight of mice during the experimental period**. Influenza (■; n = 12), smoke alone (▲; n = 8) and smoke and influenza (- ◆ -, n = 12). Mice lost weight while smoke exposure was underway, and once exposure stopped, weight was regained but this was impaired in smoke and influenza mice. Mice were first weighed 3 days before smoke exposure began and weights are expressed as mean change from this starting weight, +/- SEM. Data were analyzed by one way ANOVA with repeated measures followed by a post hoc Fisher's protected least significant difference test. The * symbol indicates a statistically significant difference in mean weight between smoke alone and smoke and influenza mice, at that time point. The # symbol indicates a statistically significant difference in mean weight between influenza alone and smoke and influenza mice at that time point.

We have previously shown that over this 4 day smoke protocol, mice lose ~6–10% of their body weight, due to nicotine-induced changes in brain neuropeptide Y which led to reduced food intake [16,21]. Figure 1 shows mice body weights. While this strain of influenza does not cause weight loss in mice, it replicates rapidly to high titres and induces a strong immune response; hence, it is considered an intermediate virulence strain [15]. No mice had any obvious illness. Both groups of smoke exposed mice regained weight as soon as smoke exposure stopped, indicating that loss of weight in smoke exposed mice was due to transient appetite suppression. However, smoke and influenza mice consistently regained less weight than smoke alone mice (p < 0.05). Influenza alone mice gained significantly more weight than smoke and influenza mice at all time points (p < 0.05). Weights of no treatment mice (not shown) continued to rise and were similar to influenza alone mice.

### Cellular influx into BALF

**Figure 2:**
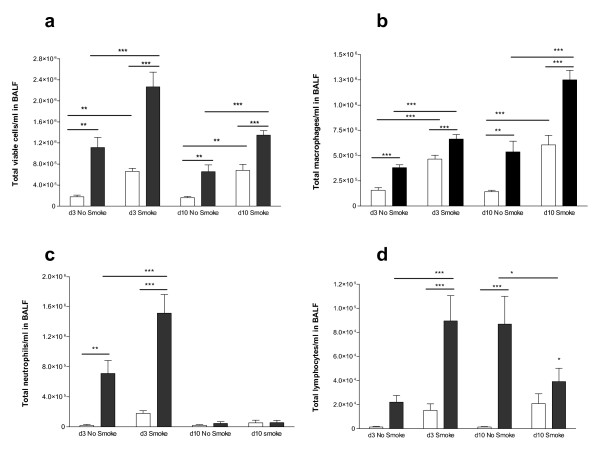
**Total and differential BALF cell counts**. Smoke increased the influx of total cells (a), macrophages (b) and neutrophils (c) into the BALF of influenza infected mice, and altered the profile of lymphocyte influx (d). Clear boxes denote mice not infected with influenza and dark boxes denote mice infected with influenza, n = 8 in all groups. Data were analyzed by two-way ANOVA, and when significance was achieved, a Bonferroni post hoc test was used. The symbol * indicates a significant change (p < 0.05); ** p < 0.01, ***p < 0.001.

Influenza caused a significant increase in total viable cells in BALF at d3, more so in smoke and influenza mice (Figure 2a). Influenza caused a macrophage influx at d3 which was higher with prior smoke exposure, while smoke alone mice at d3 and d10 had more macrophages in BALF than no treatment mice (Figure 2b). Macrophage numbers were higher in influenza alone mice at d10 than at d3, with prior smoke exposure leading to significantly more macrophages than in influenza alone mice.

Influenza caused BALF neutrophilia at d3 which was higher in smoke and influenza mice (Figure 2c). Smoke exposure prior to influenza infection was associated with an earlier peak and earlier decline of total lymphocytes in BALF, than in influenza alone mice (Figure 2d). Eosinophil numbers in BALF were consistently very low (not shown). Overall, smoke exposure generally increased inflammatory cells in BALF, even though at the d10 time point, mice had not been smoke exposed for 11 days.

To exclude the possibility that inflammation in influenza infected mice was due to cell proteins in the virus stock, separate groups of mice were intranasally inoculated with clarified supernatant of mock-infected MDCK cells, made at the same time as the virus stock, using the same media and adjusted to the same protein content. With or without prior smoke exposure, inoculation of mice with MDCK supernatant caused only a very small rise in inflammatory cells in BALF (results not shown) indicating that influenza infection caused specific, virus-induced inflammation.

### Lung virus titres

**Figure 3:**
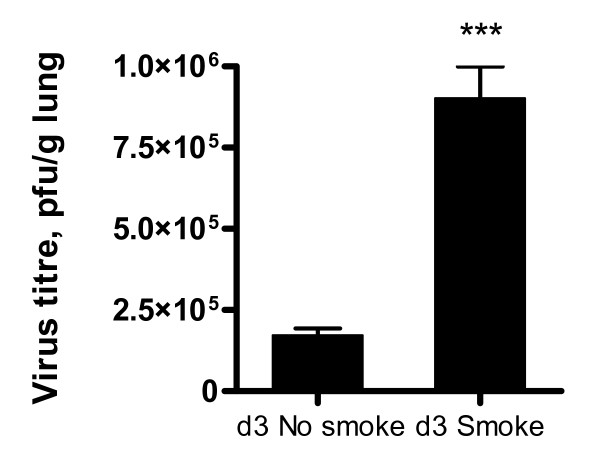
**Virus titres in mouse lung**. Titres were determined by plaque assay of individual lung homogenates in MDCK cells, n = 8 per group. Prior smoke exposure was linked to higher virus titres. Data are mean +/- SEM, ***p < 0.001, Student's two-tailed t-test. Results shown are for the d3 time point, at d10 virus was undetectable in both smoke and influenza and influenza alone mice.

These were determined by plaque assay of lung homogenates (Figure 3). Smoke and influenza mice had significantly higher virus titres at d3 than influenza alone mice. At d10 both groups of mice had no detectable virus in lungs (not shown).

### Lung proteases

**Figure 4:**
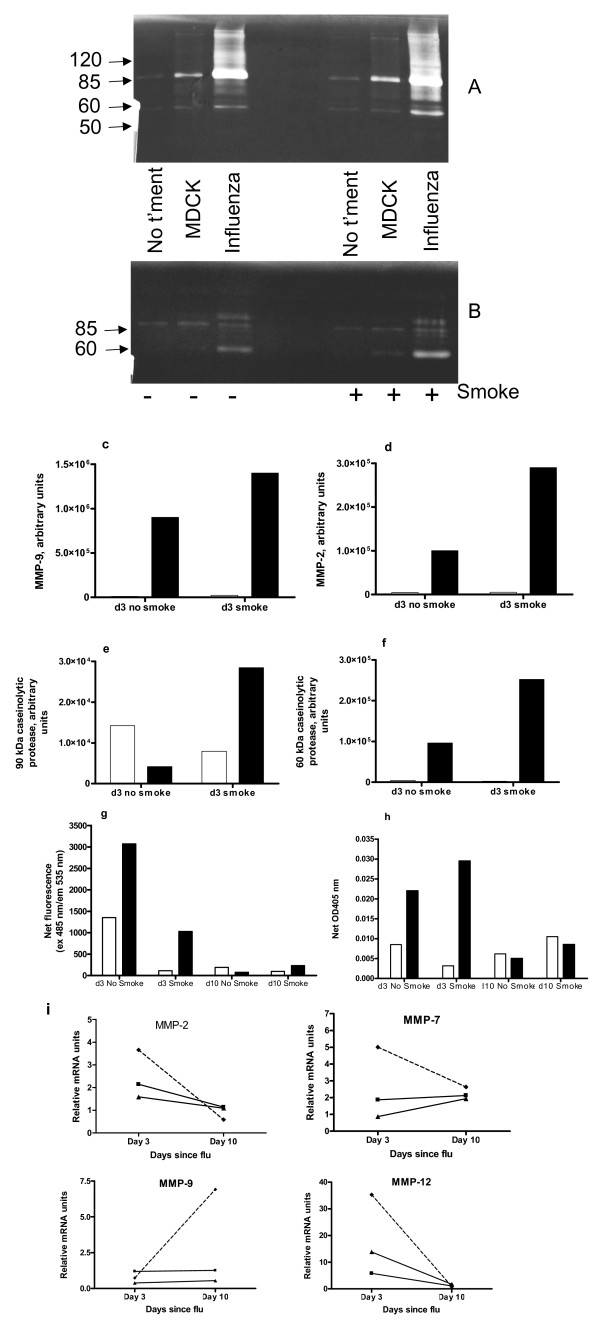
**Lung protease activity and gene expression**. In bar graphs clear boxes denote mice not infected with influenza and dark boxes denote mice infected with influenza. Panels a-h show protease activity in BALF. (a) Zymography of gelatinase activity at d3, (b) zymography of caseinolytic activity at d3 (the molecular mass of protein standards in kDa is indicated on the left side), (c) densitometry of MMP-9, (d) densitometry of MMP-2, (e) densitometry of 90 kDa caseinolytic protease, (f) densitometry of 60 kDa caseinolytic protease, (g) net gelatinase activity and (h) net serine protease activity in BALF, determined by fluorometric and colorimetric assays (respectively) in 96 well plates. BALFs of the 8 mice in each group were pooled for all of these assays. For the assays in panels g and h, this graph is representative of two assays run with the same samples, and every sample was run in triplicate. Panel i shows quantitation of relative mRNA units of proteases in mouse lung. Legend: ■ = influenza alone mice, ▲ = smoke alone and - ◆ - = smoke and influenza mice. Tissue pieces of equivalent size from lungs of the 4 mice in each treatment group were pooled prior to RNA extraction, and the cDNA was used in triplicate reactions. Fold changes are expressed relative to mRNA levels in lungs of no treatment mice.

In most respects, influenza increased protease activity in lungs and this was amplified by cigarette smoke. Zymography separates active proteases from complexes with inhibitors, indicating total active protease [22]. Major bands of gelatinase activity detected were at ~90 and 60 kDa, corresponding to MMP-9 and MMP-2 respectively [22]. Total gelatinase (Figure 4a) activity in BALF was elevated at d3 in influenza alone mice, more so with prior smoke exposure (Figures 4c and 4d). Total gelatinase activity in BALF at d10 was much lower for all groups of mice (not shown). The major bands of caseinolytic activity at d3 were at ~90 and 60 kDa (Figure 4b). Densitometry indicated that the 90 kDa protease was fairly abundant in BALF of uninfected mice, with and without smoke exposure (Figure 4e) but was most active in BALF of smoke and influenza mice at d3. This protease had the same relative abundance across groups at d10, but less overall activity (not shown). The 60 kDa caseinolytic protease was induced by influenza at d3, more so with prior smoke exposure (Figure 4f); this protease was undetectable at d10.

The fluorogenic substrate assay for net gelatinases measures predominantly MMP-2 and MMP-9, while the assay for net serine proteases mostly detects neutrophil elastase (the major serine protease in BALF). In contrast to zymography, proteases bound to endogenous inhibitors will not be detected in these assays. Compared to the influenza alone group, mice exposed to smoke and then influenza had reduced net gelatinase activity in BALF (Figure 4g) but increased net serine protease activity at d3 (Figure 4h). Smoke reduced net gelatinase activity in both virus infected and uninfected mice at d3. Gelatinase activity was low in all groups at d10. For both influenza infected and uninfected mice, smoke exposure was associated with higher net serine protease activity in BALF at d10.

Lung mRNAs encoding the gelatinases MMP-2 and MMP-9, as well as MMP-7 (matrilysin) and MMP-12 (macrophage metalloelastase) were quantitated by real time PCR (Figure 4i). MMP-2 was slightly increased in smoke and influenza mice at d3. Smoke and influenza mice had increases in gene expression of MMP-7 at d3 only and of MMP-9 at d10 only. MMP-12 mRNA levels were increased in all mice, especially smoke and influenza mice, at d3 only.

### Antiproteases

**Figure 5:**
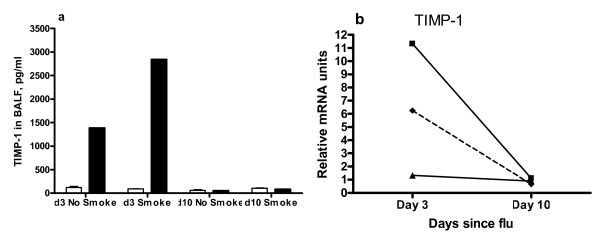
**TIMP-1 protein in BALF**. TIMP-1 was quantitated by ELISA (a) and real time PCR of lung mRNA (b). Clear boxes denote mice not infected with influenza and dark boxes denote mice infected with influenza. BALFs of the 8 mice in each group were pooled and used in duplicate ELISAs for TIMP-1. Legend in (b): ■ = influenza alone mice, ▲ = smoke alone and - ◆ - = smoke and influenza mice. Tissue pieces of equivalent size from lungs of the 4 mice in each treatment group were pooled prior to RNA extraction, and the cDNA was used in triplicate reactions. Fold changes are expressed relative to mRNA levels in lungs of no treatment mice.

TIMP-1 protein was elevated in BALF of influenza infected mice at d3, more so if mice were smoke exposed prior to influenza infection (Figure 5a). At d10, TIMP-1 was present in BALF in low amounts for all mice. In contrast, smoke exposure reduced the influenza-associated rise in lung TIMP-1 mRNA at d3 (Figure 5b). Lung mRNA levels of TIMP-2 and TIMP-3 were determined by real time PCR but all groups had little change relative to no treatment mice (not shown).

### Flow cytometry of lymphocytes

**Figure 6:**
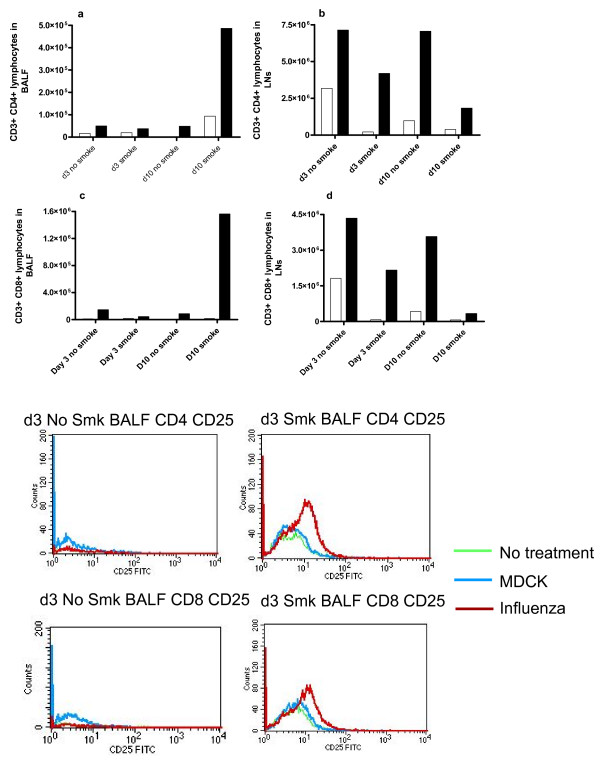
**Flow cytometry quantitation of T lymphocytes**. CD3+ CD4+ lymphocytes in (a) BALF & (b) LNs, and CD3+ CD8+ lymphocytes in (c) BALF and (d) LNs were quantitated by flow cytometry. Clear boxes denote mice not infected with influenza and dark boxes denote mice infected with influenza. The y-axis counts represent the total figure for the pooled samples of 8 mice per treatment. Figure 6(e) is a histogram presentation of CD4+CD25+ and CD8+ CD25+ cells in BALF (pooled samples of 8 mice per group), with and without smoke exposure.

Some key types of T cells were quantitated in BALF and LNs by FACS. Smoke and influenza mice had fewer CD3+ CD4+ (helper T) lymphocytes and CD3+ CD8+ (cytotoxic T lymphocytes) in their LNs, at both d3 and d10, than influenza alone mice (Figures 6b and 6d). However, at d10, smoke and influenza mice had many more CD4+ and CD8+ T cells in BALF, than influenza alone mice (Figures 6a and 6c). Smoke alone mice had more CD4+ T cells in BALF at d10 than no treatment mice.

Activation of T cells in BALF at d3 was assessed by CD25α staining [23]. In mice not subjected to smoke exposure, intensity of CD25α staining on both CD4+ and CD8+ cells in BALF was low for influenza and no treatment mice. Smoke exposed mice had greater numbers of both CD4+ and CD8+ cells that stained more intensely for CD25, particularly with influenza infection (Figure 6e). In LNs, all groups had similar CD25 staining on CD4+ and CD8+ cells, regardless of smoke exposure (results not shown).

Staining for the CD44 activation marker was done at d10. Influenza alone mice had slightly more CD4+ CD44+ and CD8+ CD44+ cells than no treatment mice in BALF, especially with smoke exposure. Neither virus nor smoke altered intensity of CD44 staining in LNs (results not shown).

### Tetramer staining

**Figure 7:**
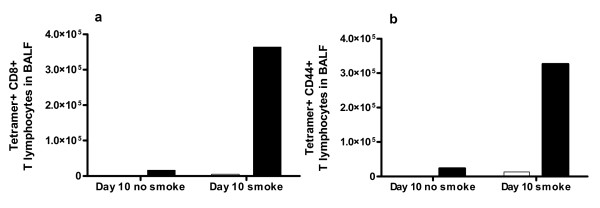
**Tetramer staining of influenza specific CD8+ T cells**. This was used to detect (a) influenza specific CD8+ T lymphocytes in BALF, and (b) influenza specific CD8+ T lymphocytes in BALF that are CD44+ positive. Clear boxes denote mice not infected with influenza and dark boxes denote mice infected with influenza. The y-axis counts represent the total figure for the pooled samples of 8 mice per treatment.

This was used to detect influenza specific cytotoxic T cells which appear in BALF around 7 days post infection [24]. Staining at d10 showed that smoke exposure before influenza caused a large increase in virus specific CD8+ T cells in BALF (Figure 7a), most of which were positive for CD44 (Figure 7b). In LNs of influenza infected mice, there were very few CD8+ tetramer+ cells, regardless of smoke exposure (not shown).

### Lung inflammatory mediators

**Figure 8:**
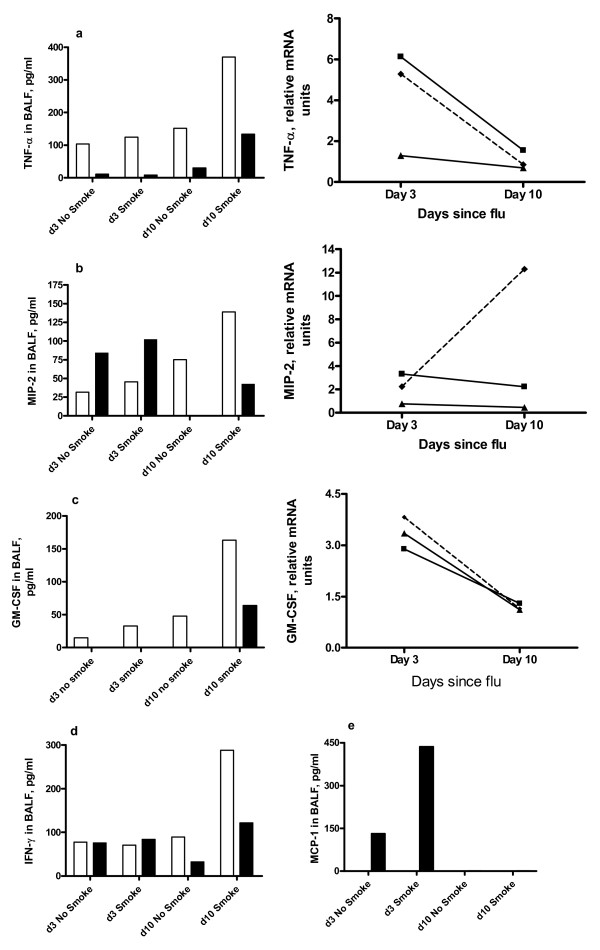
**Cytokine quantitation in BALF and lung**. Protein levels in BALF were determined by ELISA (left) while mRNA levels of the corresponding cytokine in lung were determined by real time PCR (a-c only, right). BALFs of the 8 mice in each group were pooled and used in duplicate ELISAs. Clear boxes denote mice not infected with influenza and dark boxes denote mice infected with influenza. Legend: ■ = influenza alone mice, ▲ = smoke alone and - ◆ - = smoke and influenza mice. Tissue pieces of equivalent size from lungs of the 4 mice in each treatment group were pooled prior to RNA extraction, and the cDNA was used in triplicate reactions. Fold changes are expressed relative to mRNA levels in lungs of no treatment mice. Cytokines assayed were (a) TNF-α, (b) MIP-2, (c) GM-CSF, (d) IFN-γ and (e) MCP-1.

**Figure 9:**
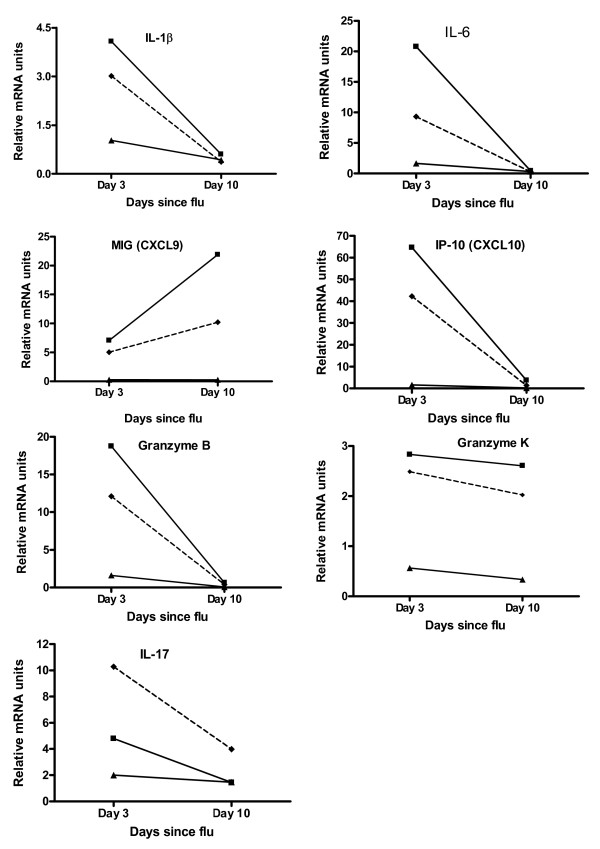
**Quantitation of relative mRNA units of inflammatory mediators in mouse lung**. ■ = influenza alone mice, ▲ = smoke alone and - ◆ - = smoke and influenza mice. Tissue pieces of equivalent size from lungs of the 4 mice in each treatment group were pooled prior to RNA extraction, and the cDNA was used in triplicate reactions. Fold changes are expressed relative to mRNA levels in lungs of no treatment mice.

ELISA was used to quantitate protein levels of inflammatory mediators in BALF, and/or real time PCR was used to quantitate mRNA levels of inflammatory mediators in lung (Figures 8 &9). Smoke exposure (without viral infection) was generally associated with higher protein levels of cytokines (e.g. TNF-α, MIP-2, GM-CSF, IFN-γ) in BALF than in no-smoke mice, especially at d10 (Figure 8a–d). We have previously shown that TNF-α mRNA in peripheral fat of smoke exposed mice was unchanged, suggesting that effects of smoke are largely confined to the lung [16]. Influenza increased d3 protein levels of the neutrophil chemoattractant MIP-2 (Figure 8b) and MCP-1 (Figure 8e), more so with prior smoke exposure. Influenza infection alone did not increase other cytokines in BALF. IL-4 protein was undetectable in any sample (data not shown).

When comparing influenza alone to smoke and influenza mice, smoke reduced the influenza-associated induction of mRNA encoding TNF-α, IL-1β, IL-6, IP-10 (interferon inducible protein 10) and granzyme B at d3, MIG (monokine induced by gamma-interferon) at d10 and granzyme K (slightly) at both d3 and d10 (Figures 8 &9). Smoke increased the influenza-associated induction of mRNA encoding IL-17 (Figure 9). The moderate induction of granzyme A mRNA in influenza infected mice was unaltered by smoke. Granzyme C mRNA was undetectable in any group (not shown).

### Histology

**Figure 10:**
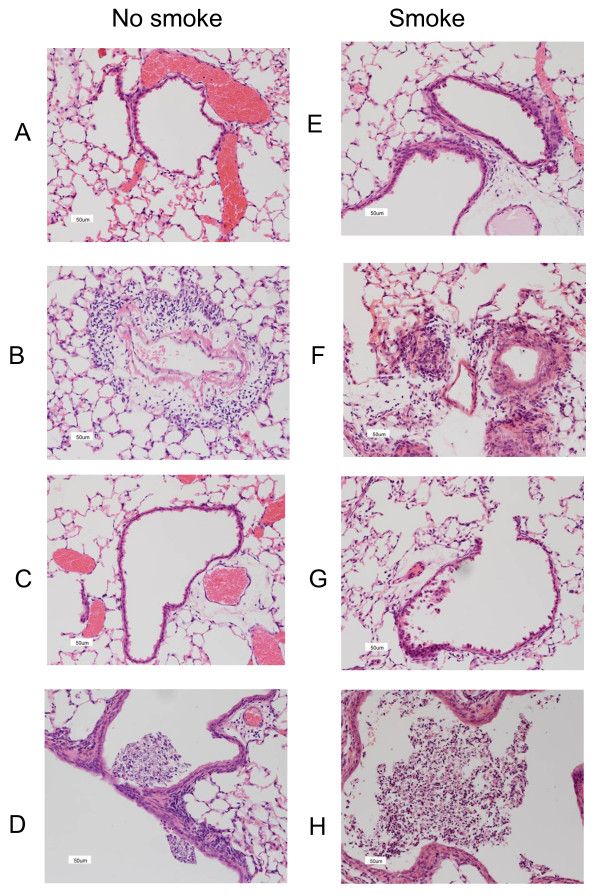
**H&E stains of lung sections from mice killed at d3**. The left panel (A-D) shows sections from mice that were not smoke exposed; these were no treatment at all mice (A and C), and influenza infected mice (B and D). The right panel (E-H) shows sections from mice that were smoke exposed; these were smoke alone mice (E and G) and smoke + influenza mice (F and H). All sections were magnified 200× and the scale bar (lower left corner of all figures) denotes 50 μm. The lungs of 4 mice from each treatment group were processed for histology and results shown were typical for the group.

Figure 10 shows H&E stains of lung sections from mice killed at d3. We have previously reported that this smoke exposure protocol causes mild lung inflammation, but not acute lung injury [12]. The top four sections (A, B, E and F) illustrate perivascular inflammation while the lower four sections (C, D, G and H) illustrate inflammatory cell exudates. Smoke alone (E, G) caused a mild inflammatory reaction comprising subtle perivascular and alveolar infiltrates of monocytes/macrophages and neutrophils. Influenza alone (B, D) caused considerable inflammation, with prominent inflammatory cell cuffing around bronchi and in the parenchyma, with some airways having small volumes of exudates containing mucus (detail revealed by periodic acid Schiff/Alcian Blue staining, not shown) and leukocytes. Inflammation was more apparent in smoke and influenza mice (F, H) with prominent mucus exudates in the airways. At d10 smoke and influenza mice had minor inflammation, but in all other groups of mice, inflammation had fully resolved (results not shown).

## Discussion

The hypothesis of this study was that short term smoke exposure of mice would activate pro-inflammatory mediators, leading to reduced viral replication and consequently, less pathology from influenza infection. However, prior smoke exposure generally led to increased inflammation and pathology after influenza infection. This was reflected in impaired weight regain once smoke exposure stopped, more total cells and macrophages in BALF at d3 and d10, more neutrophils in BALF at d3, higher viral titres at d3, generally higher protease activity in BALF and greater lung inflammation, as assessed by histology. Smoke before influenza infection led to more influenza specific cytotoxic T lymphocytes in BALF at d10. In line with other reports that cigarette smoke has both pro- and anti-inflammatory effects [10], some aspects of inflammation and pathology were reduced in mice subjected to smoke exposure before influenza infection. For example, net gelatinase activity in BALF and numbers of CD4+ and CD8+ lymphocytes in LNs were reduced in mice that were smoke exposed before influenza infection, in comparison to mice that were not smoke exposed before influenza infection.

In this study we have adopted a previously validated smoke exposure regime which achieves blood carboxyhaemoglobin levels comparable to those found in regular human smokers [25] and smoke particulate matter levels comparable to those reported by other research groups using mouse models [26,27]. It should be noted however that smoke exposure in humans is highly variable. Accordingly, there is no way to "standardize" exposure in mice. In addition to achieving carboxyhaemoglobin levels similar to smokers and particulate densities known to be relevant, our smoke exposure system also achieves exposure to nicotine levels high enough to suppress appetite [16]. Appetite regulation is a major motivation to smoke in humans. In our model there is only minor induction of systemic inflammation, as smoke did not change TNF-α transcripts in adipose tissue [16] and on smoking cessation animals immediately regain weight (Figure 1). Considered in concert with the mild lung pathology we observe in smoke alone mice, this indicates that the model is driven principally by local lung responses to smoke. The lack of acute lung injury, also observed in histopathology, is consistent with the weak systemic changes we have observed.

This novel animal model will be useful in studying the interaction of smoke, influenza and perhaps other respiratory viruses such as respiratory syncytial virus (RSV). There are only a few papers describing infection of smoke exposed mice with influenza or other respiratory viruses, and the great variation in properties of different influenza strains further complicates comparisons. The focus of older studies, such as pre-cancerous lesions in the lung [28] and effects of smoke on immune responses to a secondary heterotypic influenza infection [29] were different to the viral and immune parameters that we explored. More recently, Robbins *et al*. profiled influenza infection of smoke exposed mice [30]. Direct comparisons between their study and ours are limited as Robbins *et al*. used the C57BL/6 strain of mice, which is less susceptible to smoke [12] a different (H1N1, A/FM/1/47) strain of influenza, and a longer smoking protocol. Their trend observation that prior smoke exposure increased inflammation and worsened outcomes from high dose influenza infection was similar to our general conclusions.

Lung proteases, mainly matrix metalloproteases and the serine protease neutrophil elastase, are essential for normal immunity and repair of damaged tissue [31]. TIMPs are the main inhibitors of MMPs; TIMPs have other diverse roles in promoting cell growth and regulating apoptosis [32,33]. Proteases normally promote leukocyte extravasation, activation of inflammatory mediators, mucus secretion and products of proteolysis are chemoattractants for inflammatory cells [31]. Hence, we hypothesized that the general increase in lung proteases observed with our short term smoke exposure protocol [12] may lead to an increased antiviral response.

Influenza infected mice had raised serine protease activity in BALF which was higher with prior smoke exposure. As neutrophils are the main source of serine proteases in BALF, this was expected. It is not clear why serine protease activity in BALF of smoke alone mice was lower at d3 and increased at d10, although changes in specific antiproteases could be a factor. Zymography showed that smoke before influenza infection led to higher total activity of MMP-2, MMP-9 and caseinolytic proteases in BALF at d3. The apparent reduction in net gelatinase activity in BALF of smoke and influenza mice at d3, in comparison to influenza alone mice, is probably due to increased TIMP-1 activity. Protease activity is regulated at multiple levels, including transcription and post-translational cleavage [31], so it was interesting that prior smoke exposure altered transcriptional induction of proteases and TIMP-1 after influenza infection.

Inflammatory mediators were quantitated in BALF and lung by ELISA and real time PCR respectively, with the goal of understanding the inflammatory cell influx. Macrophages and neutrophils in particular are essential for clearance of infected and damaged tissue but their mediators may cause harm [34,35]. The rise in macrophages from d3 to d10 could be due to local macrophage proliferation and/or chemotaxis in response to matrix breakdown products [36], consistent with the induction of some proteases that we observed.

We have previously found that this short term smoke exposure protocol increased mRNA levels of many pro-inflammatory mediators in lung and/or alveolar macrophages [12]. In the current study, mRNA and protein levels for the same mediator were often not concordant; many of these inflammatory mediators may be subject to post-transcriptional regulation. Macrophage and neutrophil counts in BALF at d3 were in line with MCP-1 and MIP-2 levels (respectively). At the protein and especially at the mRNA level, many cytokines have a short half life [23]. Precise determination of cytokine mechanisms may have required more time points, and analysis of other samples such as lung homogenate or serum. It was interesting that protein amounts of the pro-inflammatory cytokines TNF-α, MIP-2, GM-CSF and IFN-γ in BALF were generally higher at both time points in BALF of smoke alone versus no treatment mice or smoke and influenza mice. This may be because smoke persistently activates some immune signalling pathways, even when smoking stops [37].

TNF-α and GM-CSF promote mobilization, activation and survival of neutrophils and macrophages [23]. TNF-α is a key factor in the lung inflammatory cell influx and matrix breakdown seen in smoke exposed mice [38]. In our study, influenza induced mRNA for these cytokines at d3, with small changes in mRNA after smoke exposure. TNF-α and GM-CSF proteins were absent or present in low amounts in BALF of influenza infected mice, except for smoke and influenza mice at d10, which may reflect production by macrophages and lymphocytes. However, GM-CSF can be hard to detect as the protein is rapidly cleared by receptor mediated internalization [39].

The higher viral titres in smoke and influenza mice at d3 may have been due to smoke reducing the interferon (IFN) response. However, quantities of IFN-α/β in BALF were low, with a small but insignificant increase in smoke and influenza mice at d3 (not shown). IFN-α/β in BALF may have already peaked and subsided by d3, and/or lung homogenate or serum may be a better sample. In contrast, IFN-γ protein in BALF showed little change in our study, aside from a rise in smoke alone mice at d10. IFN-γ is critical in antiviral immunity and smoke-induced macrophage activation [40,41]. Both CD4+ and CD8+ T cells produce IFN-γ, and smoke reduced the number of these cells in LNs of influenza infected mice at both d3 and d10, while smoke and influenza mice had very high numbers of both types of T cells in BALF at d10.

BALF counts of neutrophils and macrophages in influenza infected mice were increased by prior smoke exposure. The failure of these "extra" cells to reduce viral replication could mean that smoke impaired the function of these and perhaps other immune cells. Similarly, in human smokers numbers of alveolar macrophages are increased, yet their phagocytic ability and output of some pro-inflammatory mediators is reduced [reviewed in [10]].

CD8+ T cells are critical for clearance of respiratory viruses, but depending on the number of CD8+ T cells and overall immune status, these cells may cause severe lung pathology [42]. CD8+ T cells are critical but insufficient for protective memory responses to influenza in mice [24] and are also implicated in tissue damage in human smokers [43] and smoke exposed mice [44]. CD4+ T cells are implicated in mucus hypersecretion in smokers [45] and are important but not essential for clearance of influenza in mice [46].

We found that prior smoke exposure altered the T cell response to subsequent infection with influenza. In particular, the number of influenza specific, activated T cells in BALF at d10 was greatly increased by smoke exposure. Smoke exposure before influenza was associated with fewer CD4+ and CD8+ lymphocytes in LNs at both d3 and d10, and a large increase in both types of lymphocytes in BALF at d10. Many cell types, including CD8+ T cells, make IP-10. IP-10 has a role in homing and retention of T cells [47] and is also implicated in smoke-induced lung damage in humans [40] and mice [44], as it stimulates macrophages to release MMP-12. MIG is both made by and is a chemoattractant for T lymphocytes [23], and it also stimulates macrophages to release MMP-12 [40]. We found that smoke reduced the influenza-associated induction of lung mRNA encoding MIG at d10 and of IP-10 at d3, which could explain how smoke modified the kinetic profiles of CD4+ and CD8+ T cells. If these mRNA changes in lung led to protein changes in BALF, it would be interesting that activated CD8+ T cells were retained in BALF of smoke and influenza mice despite reduced levels of typical T cell retention mediators. As smoke also reduced mRNA expression of the T cell mediators, granzymes B and K in lungs of influenza infected mice, this could mean that CD8+ T cells of these mice have impaired function.

With this smoking protocol we previously observed increased mRNA encoding both TGF-β and IL-6 [12], which cross regulate the balance between anti-inflammatory regulatory T cells (Treg) and pro-inflammatory Th17 lymphocyte subsets. TGF-β is involved in proliferation of Treg [48] and suppression of CD8+ T cell activity by Treg [49]. Cells that are CD3+CD4+ CD25+ are typically activated T helper cells [50]. This marker profile also occurs on Treg, which also express Foxp3 and dampen inflammation [23]. Staining intensity for CD25 in BALF at d3 was slightly less in influenza alone mice (relative to no treatment mice) but higher with smoke exposure, especially after influenza infection. While we did not conclusively identify these CD25+ cells as Treg, we speculate that smoke exposure led to more Treg, which may have contributed to greater viral replication via reduced CD8+ T cell activity. This remains to be formally tested.

The precise pathological and molecular basis of how smoke modifies influenza infection is the subject of ongoing studies. Preliminary work with small group sizes and many time points strongly suggested that smoke did not simply move the kinetics of the inflammatory response (not shown). The higher virus titres at d3 in smoke and influenza mice could also be due to effects of smoke on natural killer cells. Another possible factor in the smoke-associated increase in influenza titres relates to the need for trypsin or related proteases for the cleavage of the viral haemagglutinin [6]. If this does not occur, new viral particles cannot spread to infect nearby cells. As cigarette smoke induces oxidative stress [51], which can inactivate antiproteases [52], cigarette smoke could lead to more trypsin in the lung and hence more efficient viral replication.

## Conclusion

Short term cigarette smoke exposure induces inflammatory mediators and cells known to be important in antiviral immunity. We have developed a novel mouse model for short term smoke exposure followed by infection with live influenza. We hypothesized that smoke would cause an overall activation of pro-inflammatory mediators, leading to a more efficient antiviral response. However, in most respects prior smoke exposure worsened influenza infection. This mouse model may be useful in understanding the balance of pro- and anti-inflammatory activities of smoke, and why smoke increases the incidence and severity of respiratory virus infections.

## Competing interests

The authors declare that they have no competing interests.

## Authors' contributions

RG designed and carried out experiments including mouse and virology studies, ELISAs, real time PCR and flow cytometry. RG also carried out data analysis and wrote the paper. MH was involved in mouse experiments, statistical analysis and writing of the paper. RV was involved in design and execution of mouse experiments and writing of the paper. JJ and RPJ were involved in mouse experiments. GD provided initial stocks of influenza A/Mem71 and methods for its use, and was involved in interpretation of virology experiments. ST provided tetramer reagents and methods for their use, and was involved in interpretation of flow cytometry data. KD was involved in design and interpretation of mouse experiments. GPA was involved in design and interpretation of experiments, histological analysis and writing the paper. All authors read and approved the final manuscript.
